# Intraoperative Management of Large Resuscitation-Associated Venous Air Embolism (VAE) for Emergent Neurological Surgery

**DOI:** 10.1155/2020/8868037

**Published:** 2020-06-06

**Authors:** Ryan S. D'Souza, Arnoley S. Abcejo, Matthew A. Sexton

**Affiliations:** Mayo Clinic, Department of Anesthesiology and Perioperative Medicine, Rochester, MN, USA

## Abstract

Venous air embolism (VAE) is a well-described phenomenon that may have life-threatening cardiopulmonary and neurological consequences. Accidental administration of air during resuscitation while using a rapid infuser is rare. Furthermore, there is a paucity of published data describing the intraoperative management of VAE during emergent nonseated neurological surgery. We report a 22-year-old previously healthy female who experienced a motor vehicle accident with severe facial and head trauma, and mixed subdural and epidural hematomas with an 8 mm midline shift. Computed tomography revealed significant air entrainment in the right heart and main pulmonary artery, with venous air tracking from the right axillary vein. Given her age, lack of preexisting cardiac comorbidities, hemodynamic stability, and critical cerebral herniation risk, further cardiac evaluation was deferred, and the patient was transferred to the operating room for emergent decompressive craniotomy. Intraoperatively, she experienced acute decrease in mean arterial pressure and end-expiratory carbon-dioxide with loss of pulse oximetry waveform concerning for obstructive VAE physiology. She was responsive to fluid resuscitation and epinephrine administration and did not experience any recurrence of obstructive VAE. This challenging case report describes positive neurologic and hemodynamic outcomes after resuscitation-associated VAE and cardiopulmonary collapse during emergency neurosurgery. Comprehensive evaluation of risk, urgency of procedure, and need for diagnostic monitoring and treatment should be personalized.

## 1. Background

Venous air embolism (VAE) is a well-described phenomenon which can have potentially life-threatening consequences [1]. Often unrecognized, VAEs can have widespread effects on almost every major organ system [2]. Seated neurosurgical procedures are higher risk for development of acute VAE [3, 4]. However, there are limited reports describing the intraoperative management of VAE during emergent nonseated neurological surgery. We present the intraoperative management for a patient with severe facial and head trauma after a motor vehicle accident (MVA) and a large resuscitation-associated VAE, who proceeded to the operating room (OR) for emergent decompressive craniotomy.

## 2. Clinical Case

A 22-year-old previously healthy female was the unrestrained passenger in a highway MVA. Upon impact, a mailbox penetrated the passenger front windshield and struck the patient's face, resulting in severe facial trauma. The patient was intubated in the field for a Glasgow Coma Scale score of 5. She was emergently airlifted to the hospital and, en route, was resuscitated with crystalloid and two units of whole blood.

**Figure 1:**
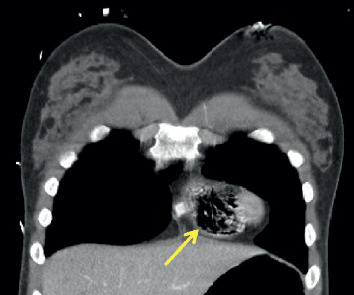
Coronal view of CT chest demonstrating significant amount of air detected in the right ventricle (arrow).

**Figure 2:**
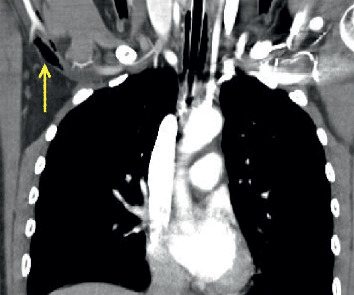
Coronal view of CT chest demonstrating air collection in the right axillary vein (arrow).

**Figure 3:**
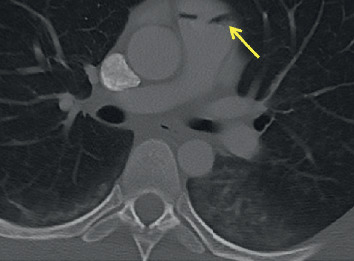
Axial view of CT chest demonstrating air trapped beneath the pulmonary valves (arrow).

The patient arrived hemodynamically unstable—tachycardic and hypotensive complicated by severe metabolic acidosis (lactate 3.42 mmol/L, reference range: 0.6–2.3 mmol/L). Her right eye was ruptured, and her left pupil was dilated to 5 mm and minimally responsive to light. Three additional units of packed red blood cells (pRBCs) and two units of fresh frozen plasma (FFP) were rapidly administered via a Level 1 rapid infuser in the right 18-gauge antecubital intravenous catheter with stabilization of her hemodynamic status. Trauma series computerized tomography (CT) revealed facial trauma with ruptured right globe, temporal bone fracture, temporomandibular joint-line fractures, and mixed subdural and epidural hematomas with an 8 mm midline shift and effacement of the anterior horn of the right lateral cerebral ventricle. A large amount of air within the right ventricle (RV) extended to the right axillary vein (Figures 1 and 2) with air collection under the pulmonary valves (Figure 3) but no immediate RV outflow tract obstruction. The air entrainment was thought to be due to transfusion of products through the Level 1 rapid infuser given that there were no pneumothoraces or air in the superior and inferior vena cava.

A multispecialty conference was held between the trauma surgery, neurosurgical, cardiac intensive care, and anesthesia teams regarding the optimal treatment plan. The concern was RV air burden may risk embolization and would compound hemodynamic collapse during emergent craniotomy. Aspiration of air via a central line was considered, but the impending risk of brain damage expedited the decision to proceed emergently to the OR for intracranial decompression. While the patient was in the CT scanner, vital signs included bradycardia (50s–60s) and occasional hypertension (systolic blood pressure reaching 150s–160s mm Hg), indicating presence of Cushing's triad. These vital signs along with physical exam findings of left dilated pupil and imaging evidence of an 8 mm cerebral midline shift were consistent with possible critically elevated intracranial pressures. With relatively stable hemodynamics but critical cerebral herniation risk, the decision was made to proceed with emergent craniotomy.

The patient arrived to the OR directly from diagnostic radiology, with heart rates between 80 and 90 beats/min and mild hypotension with systolic blood pressures of 90–100 mm Hg without any vasopressor support. She was maintained on inhaled sevoflurane at a minimum alveolar concentration <1.0 with nondepolarizing muscle relaxant. A right radial arterial line was placed. For access, the patient had a previously placed 18-gauge peripheral intravenous and intraosseous catheters. The patient was positioned in pins in the reverse Trendelenburg position. The degree of reverse Trendelenburg position was small (about 10–15 degrees) and was performed to decrease intracranial pressure and for optimum surgical visualization.

Twenty-five grams (200 mg/kg) of intravenous mannitol was administered. At this point, the patient had approximately 2–2.5 L of estimated blood loss and had received four units of red blood cells (two whole blood, two pRBCs) and four units of FFP. Since intraoperative hemoglobin (9.7 g/dl), coagulation panel, and lactate (normalized to 2.0 mmol/L) were normal, further transfusions were deferred.

Approximately 28 minutes after incision, end-expiratory CO_2_ (EtCO_2_) and mean arterial pressure abruptly fell concomitantly with loss of pulse oximetry waveform. She maintained an organized rhythm but distal pulses could not be palpated. The neurosurgeons were able to palpate intracranial pulsation; therefore, chest compressions were not initiated. Obstructive VAE physiology was assumed, and repeated intravenous epinephrine doses (10 mcg per injection) and 1 L fluid bolus were administered. After the first 30 mcg of epinephrine was administered over approximately 30 seconds, EtCO_2_ and blood pressure increased and the pulse oximetry waveform returned. An epinephrine infusion was initiated to maintain hemodynamic stability. In aggregate with hemodynamic resuscitation, the patient was immediately placed on 100% inspired oxygen and taken out of reverse Trendelenburg position to a more neutral position.

The patient remained hemodynamically stable on the epinephrine infusion and did not have a recurrence of such an event for the rest of the procedure. At the conclusion of the right hemicraniectomy and hematoma evacuation, intraoperative transesophageal echocardiography (TEE) showed hyperdynamic contractility and persistent, though nonobstructive air in the right ventricle. The intracardiac air burden was substantially less compared to the amount visualized on preoperative CT scan. There was no patent foramen ovale or regional wall abnormalities. At this point, the patient had displayed normal and stable vital signs for two hours. Thus, in the presence of hemodynamic stability in a patient without cardiac or other major comorbidities, the decision was made to proceed with the remainder of planned surgery. Furthermore, the ophthalmology and plastic surgery teams indicated that urgent surgical intervention may potentially preserve function of the patient's right eye while decreasing chance of infection.

Once the TEE probe was removed, the ophthalmology and plastic surgery teams performed a right eye enucleation, repair of brow and facial lacerations, complex repair of nasal laceration and cartilage, repair of upper and lower eyelid lacerations and reduction and fixation of numerous, complex frontozygomatic fractures, and irrigation and debridement of open wound, which collectively took 90 minutes of additional operating time.

Total OR time totaled 569 minutes. Using a goal-directed transfusion threshold of 8.0 g/dL, the patient received a total of two units of pRBCs and two units of FFP intraoperatively. Additionally, a total of 5,000 cc of crystalloid and two grams of intravenous calcium chloride were administered. Estimated blood loss for the procedure was 300 mL with 4000 mL of urine output. The epinephrine infusion was weaned off by the end of the procedure (highest dose 0.1 mcg/kg/min).

A postoperative head CT revealed expected postoperative changes with complete resolution of midline shift. The patient was then transported to the neurological intensive care unit intubated for ongoing cares. Given her age, lack of preexisting cardiac comorbidities, and resolution of her hemodynamic instability, further cardiac evaluation was deferred. She remained intubated for airway protection but was extubated within 36 hours of admission. The patient exhibited no gross neurological deficits and was able to verbally communicate shortly after extubation. She continues to progress with traumatic brain rehabilitation in the outpatient setting.

## 3. Discussion

We discuss a medically challenging emergent case wherein an otherwise healthy patient status-post MVA presented with facial trauma, subdural and epidural hematoma, and experienced hemodynamic instability from a large VAE. The VAE was likely associated with resuscitation through a Level 1 infuser. It demonstrates the clinical impact and considerations of a persistent peripheral, large-volume VAE complicating emergent craniotomy. It also highlights the consequences and management of postponing central venous aspiration to avoid delaying intracranial hematoma evacuation, threatening neurologic collapse.

VAEs may lead to multiorgan system effects including coagulopathy [1, 3, 5] and can be fatal if unrecognized [2]. High-risk procedures typically include sitting craniotomy, posterior fossa surgery, laparoscopic surgery, caesarean delivery, and central venous catheter placement/removal [6]. There have been anecdotal reports of rapid infuser-associated VAEs [7, 8]. Given the large amount of air present in the RV on initial diagnostic imaging with concurrent air tracking through the right axillary vein, we posit the rapid infuser was the source of VAE. The two most important factors in determining morbidity and mortality from VAE are the volume and rate of air entrainment [9]. It is plausible that the infuser was incorrectly primed. We likely observed an intraoperative hypotensive crisis secondary to peripheral translation of air into the RV, resulting in airlock obstruction. That air from peripheral veins likely remained stationary due to peripheral valves. Another possible source may include air entrainment through the maxillary and zygomatic bone fractures [10, 11]. While it is also plausible that the VAE may have originated from the neurosurgical incision and/or durotomy, this is unlikely given the timing of VAE-associated hemodynamic compromise and no iatrogenic injury to intracerebral large vessels.

There were several key decision points in managing this patient. The first decision point came after radiographic imaging revealed a significant quantity of RV air. The question became whether this patient was stable for an interventional radiologic procedure prior to neurosurgery to remove the entrained air. With only modest hypotension but critical cerebral herniation risk, the collective decision was made to proceed with emergent craniotomy. If the patient was hemodynamically unstable and unresponsive to fluid administration or vasopressor support, we would have postponed neurosurgery and intervened with central line placement for air aspiration and cardiopulmonary resuscitation as needed. The next decision point presented intraoperatively to decide if a central line should be placed to aspirate air either through the line itself or through a pulmonary artery catheter. Although there is evidence to suggest CT-guided aspiration of air can be life-saving, there is limited evidence to suggest a benefit without imaging guidance from CT or echocardiography [6, 8, 12]. Additionally, the placement of another central line catheter is itself an independent high-risk procedure for VAE [6]. Furthermore, the risk of exacerbating intracranial pressure from Trendelenburg positioning during central line placement outweighed its placement. While a long-arm central line was considered, we ultimately decided not to place a central line at the expense of delaying surgery. Another decision point involved optimal surgical positioning. A small degree of reverse Trendelenburg positioning was employed to assist in decreasing intracranial pressure and optimizing surgical visualization. Although seated or reverse Trendelenburg position may contribute to increased risk for air entrainment into the circulation, there is no clear evidence that the angle of the head with respect to the heart contributes to any exacerbation of air-related RV failure. Studies have demonstrated no difference in air entrainment in the right atrium and in the pulmonary artery in the seated position versus supine position [4, 13].

We then discussed the utility of other recommended intraoperative monitoring tools and treatment options, including TEE, precordial Doppler, patient positioning, vasopressor support, and hyperbaric oxygen therapy [6, 14]. Given the confirmed presence of air within the RV from preoperative CT, we felt a precordial Doppler would yield little clinical benefit. While we utilized intraoperative TEE after the hemicraniectomy, this was delayed due to the urgency of beginning the surgery, absence of comorbidities, and stable hemodynamics. Our intraoperative use of epinephrine was selected due to its combined inotropic, chronotropic, and peripheral vasoconstrictor effects. Postoperatively, given the patient's stable hemodynamics and relatively unremarkable postoperative head CT, we did not believe additional interventions such as hyperbaric oxygen therapy were indicated.

If the patient continued to experience hemodynamic instability despite epinephrine administration, we would discuss this immediately with the neurosurgery team and whether it would be safe to place the patient in a neutral or Trendelenburg position, being cognizant of potentially worsening the intracranial hematoma and ICP. If repositioning was not possible, we would have considered the Durant maneuver (partial left lateral decubitus position). Additional vasopressor agents may be considered including dobutamine [15] and norepinephrine [16], with the goal of optimizing myocardial perfusion, relieving air entrainment, and providing RV inotropy. Consideration of flooding the surgical field with saline or placing soaked dressings may be beneficial if the potential source of air entrainment was via cerebral vessels. While studies mention success rates of only 6–16% from aspirating air centrally [6, 17, 18], we would consider placing a multiorificed central line for air aspiration through the internal jugular vein if the patient could be safely placed in Trendelenburg position. If repositioning was not possible, we would place a long-arm central line through the antecubital vein and would guide the catheter either using chest X-ray guidance or an electrocardiogram lead attached to the catheter [18]. In the event of cardiac standstill or severe persistent hypotension, we would initiate cardiopulmonary resuscitation with closed chest compressions, which may aid in hemodynamics and potentially force air out of the pulmonary outflow tract and through the pulmonary vessels improving forward flow [19, 20].

## 4. Conclusion

This challenging case report describes positive neurologic and hemodynamic outcomes after resuscitation-associated VAEs and cardiopulmonary collapse during emergent neurosurgery. While few reports of rapid infuser-associated VAEs have been described, no prior reports have occurred during emergent neurosurgery where risk-benefit analysis determined the feasibility of pursuing emergent surgery while postponing interventional management of VAE in a hemodynamically stable patient. Comprehensive evaluation of risk, urgency of procedure, and need for diagnostic monitoring and treatment should be personalized. We acknowledge this patient's positive outcome was not solely due to the intraoperative management by the anesthesia team but can also be attributed to the skill of the surgical teams, patient's age, and absence of comorbidities.

## Data Availability

Data are available on request to Matthew Sexton (sexton.matthew@mayo.edu).
